# *Angelica sinensis* Polysaccharide and *Astragalus membranaceus* Polysaccharide Accelerate Liver Regeneration by Enhanced Glycolysis via Activation of JAK2/STAT3/HK2 Pathway

**DOI:** 10.3390/molecules27227890

**Published:** 2022-11-15

**Authors:** Xu-Dong Wen, Yao-Lei Zhang, Ling Yang, Zhen Ye, Guo-Chuan Fu, Yong-He Hu, Tao Pan, Qiao-Bo Ye

**Affiliations:** 1 Department of Gastroenterology, Chengdu Integrated TCM&Western Medicine Hospital, Chengdu University of Traditional Chinese Medicine, Chengdu 610059, China; 2 School of Materials Science and Engineering, Southwest Jiaotong University, Chengdu 610031, China; 3 School of Clinical Medicine, Chengdu University of Traditional Chinese Medicine, Chengdu 610032, China; 4 College of Basic Medical Sciences, Chengdu University of Traditional Chinese Medicine, Chengdu 610032, China

**Keywords:** *Angelica sinensis* polysaccharide, *Astragalus membranaceus* polysaccharide, hexokinase 2, glycolysis, JAK2/STAT3 pathway, liver regeneration

## Abstract

The promotion of liver regeneration is crucial to avoid liver failure after hepatectomy. *Angelica sinensis* polysaccharide (ASP) and *Astragalus membranaceus* polysaccharide (AMP) have been identified as being associated with hepatoprotective effects. However, their roles and specific mechanisms in liver regeneration remain to be elucidated. In the present study, it suggested that the respective use of ASP or AMP strikingly promoted hepatocyte proliferation in vitro with a wide range of concentrations (from 12.5 μg/mL to 3200 μg/mL), and a stronger promoting effect was observed in combined interventions. A significantly enhanced liver/body weight ratio (4.20%) on day 7 and reduced serum transaminase (ALT 243.53 IU/L and AST 423.74 IU/L) and total bilirubin (52.61 IU/L) levels on day 3 were achieved by means of ASP-AMP administration after partial hepatectomy in mice. Metabonomics showed that differential metabolites were enriched in glycolysis with high expression of beta-d-fructose 6-phosphate and lactate, followed by significantly strengthened lactate secretion in the supernatant (0.54) and serum (0.43) normalized to control. Upon ASP-AMP treatment, the knockdown of hexokinase 2 (HK2) or inhibited glycolysis caused by 2-deoxy-d-glucose decreased hepatocyte proliferation in vitro and in vivo. Furthermore, pathway analysis predicted the role of JAK2/STAT3 pathway in ASP-AMP-regulated liver regeneration, and phosphorylation of JAK2 and STAT3 was proven to be elevated in this promoting process. Finally, downregulated expression of HK2, an attenuated level of lactate secretion, and reduced hepatocyte proliferation were displayed when STAT3 was knocked out in vitro. Therefore, it can be concluded that ASP-AMP accelerated liver regeneration and exerted a hepatoprotective effect after hepatectomy, in which the JAK2/STAT3/HK2 pathway was actively involved in activating glycolysis.

## 1. Introduction

Liver failure is a potentially fatal complication after liver resection, with an incidence of about 7% in patients with healthy parenchyma and as high as 30% in those with cirrhosis [1,2]. Although the liver possesses an intrinsic regenerative capacity, it is challenging for the host to survive after major liver resection, with there being a high mortality rate [3]. Improving the liver’s capacity to regenerate is crucial, as demonstrated by accumulating evidence surrounding this highly complex process [4]. Currently, there are no clinically available therapeutic agents that enhance liver regeneration, especially in a post-liver-resection setting [5].

To promote liver regeneration, we attempt to search for natural products from traditional Chinese medicines as an abundant resource to be utilized in drug development [6]. The Danggui Buxue Decoction (DBD) is composed of *Angelica sinensis* and *Astragalus membranaceus* at the ratio of 1:5 and has been widely used as a Chinese herbal medicine and functional food for thousands of years [7,8]. The DBD has also been proven to exert a protective effect on hepatic fibrosis [9] and have tissue regeneration capability [10]. As major active ingredients from *Angelica sinensis* and *Astragalus membranaceus*, *Angelica sinensis* polysaccharide (ASP) and *Astragalus membranaceus* polysaccharide (AMP) possess a variety of beneficial pharmacological activities towards the liver [11,12]. Evidence indicates that ASP and/or AMP exert anti-fibrosis [12] and hepatoprotection [13,14] activities. These results strongly support the potential for liver regeneration promotion by means of the combined use of ASP and AMP (ASP-AMP).

The liver maintains glucose homeostasis, in which gluconic biomolecules are dynamically regulated to provide energy as well as the anabolic molecules required for liver regeneration [15]. Metabonomics indicates that liver metabolites change remarkably before and after hepatectomy [16]. Interestingly, polysaccharides have been proven to activate liver glycometabolism-related signaling pathways in diabetes mellitus rats [17]. AMP showed beneficial effects in lowering blood glucose and triglyceride levels [18]. Meanwhile, during the regeneration process, hexokinase 2 (HK2) is actively involved in glycolysis, the inhibition of which leads to reduced proliferation of hepatic cells [19]. These observations suggest that HK2-mediated glycometabolism might be directly related to ASP-AMP-assisted liver regeneration. In addition, ASP and AMP have recently been proposed to be involved in various pathway regulations via triggering a variety of molecular responses in the liver [11,20]. ASP induces IL-22 secretion in liver tissue via the activation of the signal transducers and activators of transcription (STAT3) pathway to attenuate liver fibrosis [21]. AMP diminished the formation of free radicals and cytokines by the downregulation of STAT3 expression [22]. To date, the regulating effect of ASP and AMP on liver regeneration has not been fully elucidated, and the potential mechanism in metabolic alternations remains to be explored.

**Scheme 1:**
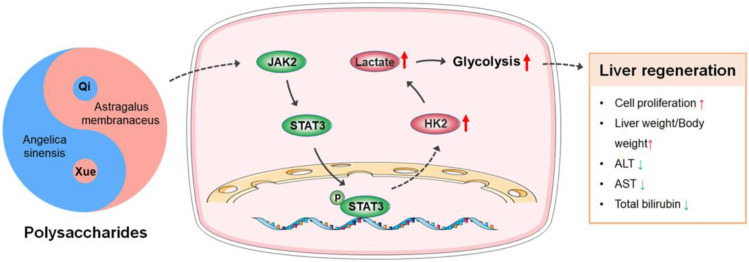
Schematic illustration of combined use of *Angelica sinensis* polysaccharide and *Astragalus membranaceus* polysaccharide promoting liver regeneration via the activation of the JAK2/STAT3/HK2 pathway and enhanced glycolysis.

The present study aimed to reveal the potential mechanism by which ASP-AMP promoted liver regeneration (Scheme 1). First, the effect of ASP and/or AMP on cell proliferation was evaluated in vitro and in vivo. Second, metabonomics was performed on hepatocytes and liver tissue treated with ASP-AMP to investigate the differential metabolites. Finally, the predicted pathways from network pharmacology were verified and the specific mechanism by which ASP-AMP promoted liver regeneration was analyzed.

## 2. Results

### 2.1. ASP-AMP Promoted Hepatocytes Proliferation In Vitro

**Figure 1:**
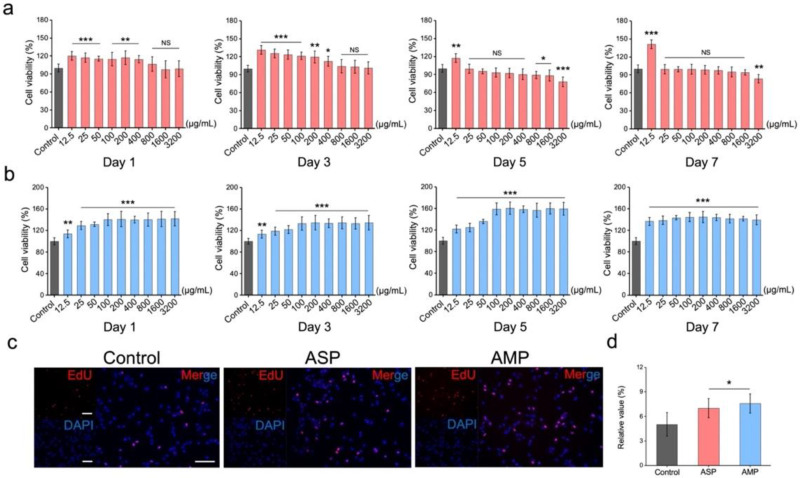
*Angelica sinensis* polysaccharide (ASP) and *Astragalus membranaceus* polysaccharide (AMP) promoted proliferation of NCTC clone 1469. The cell viability of hepatocytes treated by various concentrations of ASP (**a**) and AMP (**b**) for 7 days, which was determined by Alamar Blue Cell Viability Reagent (*n* = 3). (**c**) Click-iT EdU proliferation staining of hepatocytes treated with ASP and AMP for 3 days (scale bar = 100 µm). (**d**) Analysis of relative expression rates of positive EdU staining (*n* = 3). * *p* < 0.05, ** *p* < 0.01, and *** *p* < 0.001.

To confirm the combined effect of ASP and AMP on hepatocytes, two normal liver cell lines were first treated with ASP or AMP with different concentrations. NCTC clone 1469 was cultured with ASP at concentrations ranging from 12.5 μg/mL to 3200 μg/mL for 7 days. The ASP (12.5 μg/mL) significantly enhanced the proliferation of NCTC clone 1469 (*p* < 0.01), but higher concentrations did not promote or even reduce the proliferation from day 1 to day 7 (Figure 1a). Meanwhile, significant differences were observed in AMP-treated groups on day 1, day 3, day 5, and day 7 (*p* < 0.001, Figure 1b). The ability of AMP to promote proliferation improved with the increase in concentration, and this promoting ability reached its peak when the concentration was 100 μg/mL. Furthermore, the promoting effect at the optimal concentration (ASP 12.5 μg/mL and AMP 100 μg/mL) was verified by the EdU assay, and it demonstrated that the ratios of red to blue points were significantly higher in ASP (7.01%) and AMP (7.58%) groups than that in the control group (5.03%) on day 3 (*p* < 0.05, Figure 1c,d). Simultaneously, the proliferation effect of ASP or AMP was verified in BRL-3A. Similar to the results in NCTC clone 1469 in Figure 1a, a relatively low dose of ASP (100 μg/mL) had the most promoting effect on BRL-3A on day 3, day 5, and day 7 (*p* < 0.01, Figure S1A, see Supplementary Materials). With the increase in ASP concentration (from 100 μg/mL to 3200 μg/mL), inhibited cell viability was observed on day 5 and day 7 in BRL-3A. AMP with a concentration of 200 μg/mL began to work, and it exerted the greatest promoting function when the concentration reached 800 μg/mL (*p* < 0.05, Figure S1b). The EdU assay indicated that the number of hepatocytes increased more rapidly in the ASP (6.41%) and AMP (6.78%) groups than that in the control group on day 3 (4.53%) (*p* < 0.001, Figure S1c,d). These results reveal that respective use of ASP and AMP at specific concentrations could promote hepatocyte proliferation.

**Figure 2:**
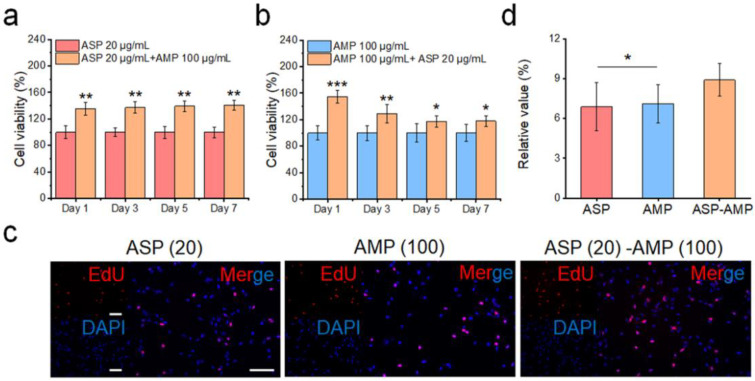
Combined treatment with both ASP and AMP enhanced the proliferation of NCTC clone 1469 cells. (**a**) The cell viability of hepatocytes treated with a combination of ASP and AMP compared to the separate use of ASP (**a**) or AMP (**b**) for 7 days (*n* = 3). (**c**) Click-iT EdU proliferation staining of hepatocytes treated with ASP, AMP, and ASP-AMP for 3 days (scale bar = 100 µm). (**d**) Relative expression rates of positive EdU staining (*n* = 3). * *p* < 0.05, ** *p* < 0.01, and *** *p* < 0.001.

Furthermore, representative concentrations of ASP and AMP were selected in terms of the promoting effect presented in Figure 1 and Figure S1. NCTC clone 1469 cells that were cultured with both ASP (20 μg/mL) and AMP (100 μg/mL) proliferated significantly faster than cells solely cultivated with ASP at a concentration of 20 μg/mL (*p* < 0.01, Figure 2a) or AMP at a concentration of 100 μg/mL (*p* < 0.05, Figure 2b). Consistent with the results of cell viability in Figure 2a,b, EdU-positive hepatocytes were observed at a rate of 8.91% in the ASP-AMP group (20 μg/mL of ASP, 100 μg/mL of AMP, Figure 2c,d). The relative value in the ASP-AMP group was significantly higher than that in ASP or AMP group (*p* < 0.05). Meanwhile, ASP with a concentration of 160 μg/mL and AMP with a concentration of 800 μg/mL were chosen to verify the promoting effect of ASP-AMP on proliferation in BRL-3A cells (Figure S2). Significantly promoted cell proliferation by ASP-AMP was observed via cell viability analysis (*p* < 0.05, Figure S2a,b), as well as in the EdU assay results (*p* < 0.05, Figure S2c,d). It was demonstrated the upon the concentration above, combined use of ASP and AMP at a ratio of 1:5 enhanced the proliferation-promoting effect compared to their separate use. 

### 2.2. ASP-AMP Accelerated Liver Regeneration and Reduced Liver Injury after Hepatectomy In Vivo

**Figure 3:**
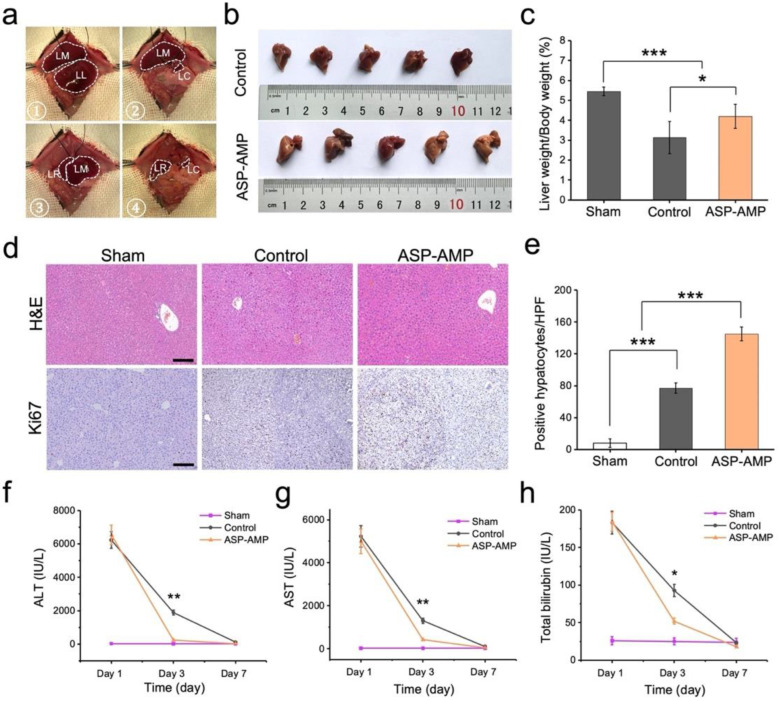
ASP-AMP enhanced liver regeneration after partial hepatectomy. (**a**) Schematic representation of 70% hepatectomy in mice. (**b**) The view of isolated liver tissues at day 7 (*n* = 5). (**c**) Ratio of liver weight to body weight 7 days after hepatectomy (*n* = 5). (**d**) Representative H&E and Ki67 staining of liver sections 3 days after hepatectomy (scale bar = 200 µm). (**e**) The quantification of Ki67 staining of liver sections 3 days after hepatectomy. Plasma ALT (**f**), AST (**g**), and total bilirubin (**h**) levels in mice 1 day, 3 days, and 7 days after hepatectomy with ASP-AMP intervention (*n* = 3). LM, liver middle lobe; LL, liver left lobe; LR, liver right lobe; LC, liver caudate lobe. ALT, alanine aminotransferase; AST, aspartate aminotransferase. * *p* < 0.05, ** *p* < 0.01, and *** *p* < 0.001.

As shown in the schematic diagram of 70% hepatectomy in mice, after median incision (①) the left (②) and middle (③) lobes of livers were resected in sequence, and the right and caudate (④) lobes of livers were left in residue (Figure 3a). After hepatectomy, Balb/c mice treated with ASP-AMP for 7 days were sacrificed, and after the harvest of the isolated liver tissue, a larger volume was displayed in the ASP-AMP group (Figure 3b). A higher liver to body weight ratio was observed in the ASP-AMP group (4.20%), being 1.34 times higher than that of control group (*p* < 0.05, Figure 3c). Meanwhile, this ratio in the Sham group was 5.44%, indicating that the residual liver mass in the ASP-AMP group increased to 77.21% of the unresected liver. H&E analysis of the liver tissues showed no nuclear condensation or fragmentation in either group (Figure 3d). Strikingly, the ASP-AMP group exhibited increased hepatocyte proliferation after hepatectomy, as evidenced by increased Ki67 staining when compared with the control and sham groups (*p* < 0.001, Figure 3d,e). ASP-AMP-treated mice displayed a potent reduction in plasma levels of alanine aminotransferase (ALT, 243.53 IU/L), aspartate aminotransferase (AST, 423.74 IU/L), and total bilirubin (52.61 IU/L) three days after hepatectomy, demonstrating the hepatoprotection of liver injury (*p* < 0.05, Figure 3f–h).

### 2.3. ASP-AMP Enhanced HK2 Involved Glycolysis to Promote Liver Regeneration

**Figure 4:**
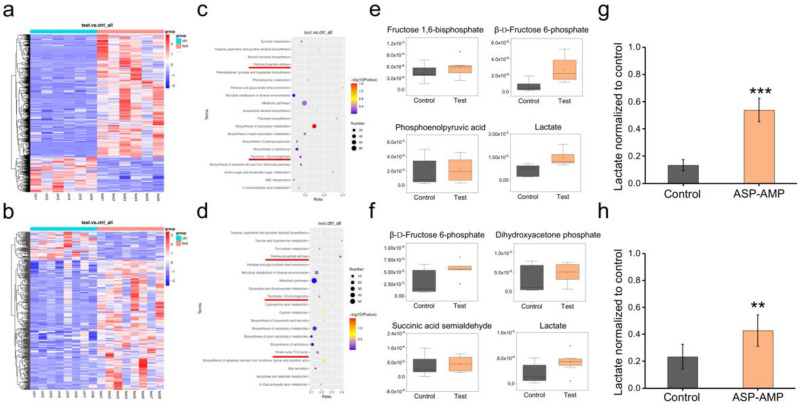
Differential metabolites found by metabolomics analysis and metabolic pathway analysis in NCTC clone 1469 cells (*n* = 6) and liver tissue (*n* = 8) after hepatectomy treated with ASP-AMP. Clustering heat map of total differential metabolites in hepatocytes (**a**) and liver tissue (**b**). Red refers to upregulated and blue refers to downregulated metabolites. KEGG pathway enrichment bubble diagram in hepatocytes (**c**) and liver tissue (**d**). The top 20 in terms of metabolite numbers are displayed, including glycolysis/gluconeogenesis, the citrate cycle (TCA cycle), and the pentose phosphate pathway. Six predictive metabolites related to glycometabolism are shown for hepatocytes (**e**) and liver tissue (**f**). Relative abundances were log10-transformed. Data were generated by LC-UHPLC. Lactate measurement in the hepatocyte supernatant (**g**) and mouse serum (**h**). Ctrl refers to the control group, and test refers to the experimental group treated with ASP-AMP for 3 days. ** *p* < 0.01, and *** *p* < 0.001.

To detect the variations of metabolites in ASP-AMP-promoted liver regeneration, metabonomics between the ASP-AMP treated group (test) and control group (Ctrl) was performed. A hierarchical clustering analysis was executed on all of the different metabolites, and the relative quantitative values of the difference metabolites were normalized, converted, and clustered. It can be intuitively observed that most of the metabolites were highly expressed in the ASP-AMP-treated groups (Figure 4a,b). These results are consistent with those in the volcano maps (Figure S3a,b). The enriched and differentially expressed metabolites in the KEGG pathway analysis are presented in bubble charts (top 20) in Figure 4c for hepatocytes and Figure 4d for liver tissue. The differential metabolites of the ASP-AMP intervention group and control group were enriched in glycolysis/gluconeogenesis, the citrate cycle (TCA cycle), and the pentose phosphate pathway (red lines). Two metabolites (beta-d-fructose 6-phosphate and lactate) with a *p* value < 0.05, and four metabolites (fructose 1, 6-bisphosphate, phosphoenolpyruvic acid, dihydroxyacetone phosphate, and succinic acid semialdehyde) with moderate elevation related to glycometabolism were displayed (Figure 4e,f). These results lay the foundation for understanding the ASP-AMP-intervened metabolic pathways and differential metabolites. Furthermore, it was verified in the hepatocyte supernatant (Figure 4g) and serum (Figure 4h) that ASP-AMP improved the lactate secretion (*p* < 0.01).

**Figure 5:**
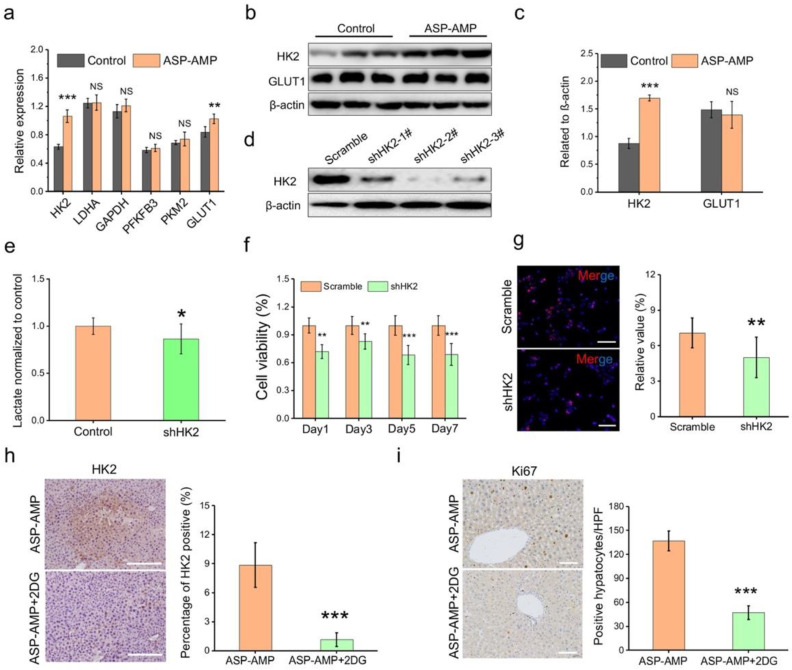
ASP-AMP accelerated the hexokinase 2 (HK2) involved glycolysis to promote liver regeneration. (**a**) RT-PCR analysis of rate-limiting enzymes related to glycolysis. (**b**) Western blot analysis for HK2 and GLUT1 after ASP-AMP treatment in NCTC clone 1469 cells (*n* = 3). (**c**) Relative protein was quantitatively expressed by densitometric analysis and is shown as the fold change relative to β-actin. (**d**) Efficiency of knockdown of HK2 in NCTC clone 1469 cells. (**e**) Lactate levels in supernatant of HK2 knockdown NCTC clone 1469 cells (*n* = 3). (**f**) The cell viability of HK2 knockdown NCTC clone 1469 cells treated with a combination of ASP and AMP for 7 days (*n* = 3). (**g**) Click-iT EdU proliferation staining of HK2 knockdown NCTC clone 1469 cells treated with ASP-AMP for 3 days and the relative expression rates of positive EdU staining (Scale bar = 100 µm). (**h**) Representative images of IHC staining for HK2 in the liver tissue of mice after hepatectomy when treated with ASP-AMP or ASP-AMP+2DG for 3 days and the analysis of percentage of HK2 positive area (Scale bar = 100 µm). (**i**) Ki67 staining of liver sections with 3 days of ASP-AMP+2DG after hepatectomy and the quantification of positive cells (scale bar = 100 µm). * *p* < 0.05, ** *p* < 0.01, and *** *p* < 0.001.

The results above reveal that lactate-assisted glycolysis was actively involved in the process of ASP-AMP promoting liver regeneration. However, the exact enzyme dominating the process remains unclear. To elucidate the mechanism by which ASP-AMP regulates liver regeneration, several genes of rate-limiting enzymes demonstrated to be modulated in glycolysis were analyzed, including HK2, lactate dehydrogenase (LDHA), glyceraldehyde-3-phosphate dehydrogenase (GAPDH), 6-phosphofructo-2-kinase/Fructose-2,6-biphosphatase 3 (PFKB3), pyruvate kinase (PKM2), and GLUT1. ASP-AMP-administered NCTC clone 1469 cells demonstrated activated HK2 and GLUT1 at transcription level (*p* < 0.01, Figure 5a). Subsequently, we verified the protein expression of HK2 and GLUT1, and HK2 was upregulated, while the regulation of GLUT1 expression was insignificant (Figure 5b,c). To assess the impact of HK2 on lactate secretion and growth of hepatocytes, knockdown of HK2 in NCTC clone 1469 cells was performed by using an shRNA specific to HK2 (HK2 shRNA#2) (Figure 5d). Knockdown of HK2 decreased the lactate secretion in the supernatant (*p* < 0.05, Figure 5e), and inhibited the proliferation of NCTC clone 1469 cells for 7 days (*p* < 0.01, Figure 5f,g). The IHC staining of HK2 was lowly expressed in the ASP-AMP+2DG group compared with the ASP-AMP group (*p* < 0.001, Figure 5h), as well as the Ki67 staining (*p* < 0.001, Figure 5i). These results confirm that ASP-AMP accelerated liver regeneration via HK2-assisted glycolysis in vitro and in vivo.

### 2.4. ASP-AMP Activated JAK2/STAT3 Signaling Pathway to Increase the Liver Regeneration

**Figure 6:**
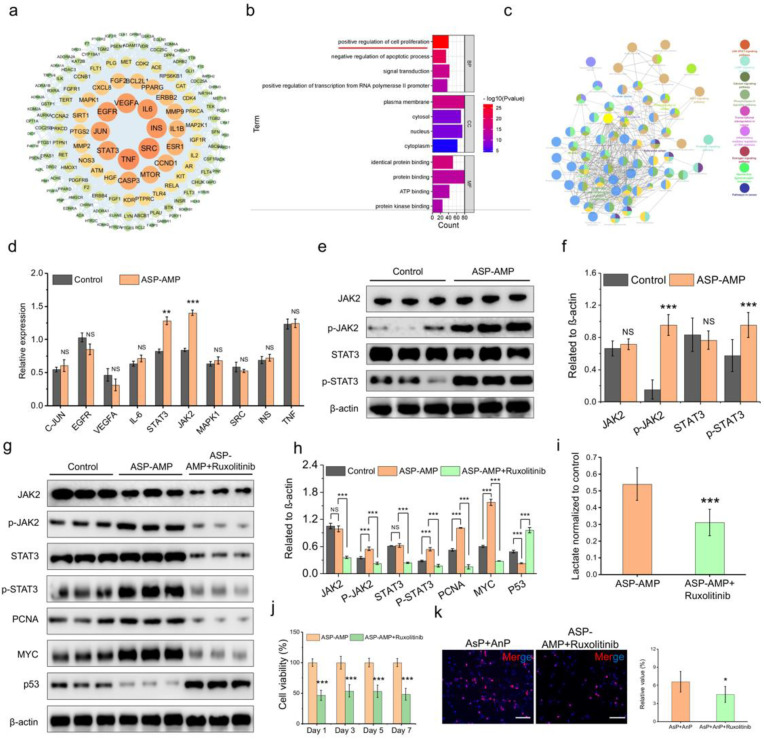
Activation of the JAK2/STAT3 signal pathway dominated the ASP-AMP-promoted liver regeneration. (**a**) PPI network of the common targets of drugs and liver regeneration. According to the degree value, the nodes were arranged from large to small and the color of the node changes from orange to yellow to green. (**b**) GO enrichment analysis. The top four terms in Biological Process, Cellular Component and Molecular Function are displayed. (**c**) The KEGG signaling pathway of ASP and AMP promoting liver regeneration. (**d**) RT-PCR analysis of predictive target factors in ASP-AMP promoted liver regeneration (*n* = 3). (**e**) The expression of JAK2, p-JAK2, STAT3, and p-STAT3 in hepatocytes were analyzed by Western blot (*n* = 3). (**f**) Densitometric analysis of grey value related to β-actin expression. (**g**) Pharmacologically inhibiting JAK2 downregulated the expression of p-STAT3, MYC, and PCNA, and upregulated p53 (*n* = 3). (**h**) Quantitative analysis of protein expression related to β-actin. (**i**) Lactate expression in the supernatant of hepatocytes with ASP-AMP and/or ruxolitinib treatment for 3 days (*n* = 3). (**j**) Cell viability of NCTC clone 1469 cells treated with ASP-AMP and/or ruxolitinib for 7 days (*n* = 3). (**k**) Click-iT EdU proliferation staining of hepatocytes treated with ASP-AMP and/or ruxolitinib for 3 days and relative expression rates of positive EdU staining (scale bar = 100 µm). * *p* < 0.05, ** *p* < 0.01, and *** *p* < 0.001.

To elucidate the potential targets and molecular mechanisms of ASP-AMP-promoted liver regeneration, network pharmacology and bioinformatics were performed. The results show that ASP and AMP obtained 321 and 156 potential targets, respectively, in which a total of 417 targets were involved. A total of 1131 proteins were selected as the potential targets related to liver regeneration (Figure S4a). According to the above results, 417 putative targets of drugs (ASP and AMP) and the 1131 liver regeneration-related targets were imported to TBtools 1.038 to obtain 145 common targets (Figure S4b). Then, a PPI network reflected the function of the targets in liver regeneration and discovered the interactive effects (Figure 6a). Enrichment analysis involved GO and KEGG pathway analysis. We selected the top four GO items according to *p* values and counted 145 genes using bioinformatics (Figure 6b). For GO biological processes, the targets were highly enriched in the positive regulation of cell proliferation (GO: 0008284) in Biological Process (red line). As for pathway analysis, the JAK/STAT signaling pathway played a significant role in promoting liver regeneration (Figure 6c).

According to the above predicted targets in Figure 6a, transcription levels of genes including C-JUN, epidermal growth factor receptor (EGFR), vascular endothelial growth factor A (VEGFA), interleukin 6 (IL-6), STAT3, JAK2, mitogen-activated protein kinase 1 (MAPK1), sarcoma gene (SRC), insulin, and tumor necrosis factor (TNF) were detected after the ASP-AMP treatment. STAT3 and JAK2 were upregulated in NCTC clone 1469 cells (Figure 6d). Western blot displayed that ASP-AMP increased phosphorylation of JAK2 and STAT3 (Figure 6e,f). Accordingly, associated downstream signaling factors, such as PCNA and MYC, were upregulated, and p53 was downregulated (Figure 6g,h). To verify that the JAK2/STAT3 pathway was the main pathway participating in the regulation of the liver by ASP-AMP, ruxolitinib was used to inhibit JAK2 activity pharmacologically (Figure 6g,h). The protein level of STAT3 and the phosphorylation of STAT3, PCNA, MYC, and p53 were reversed. Lactate in the supernatant decreased accordingly in the ASP-AMP+ruxolitinib group (*p* < 0.001, Figure 6i). The inhibition of JAK2 reduced cell proliferation for 7 days (*p* < 0.05, Figure 6j–l). These results demonstrate that JAK2/STAT3 dominated the process of the ASP-AMP-accelerating liver regeneration.

### 2.5. STAT3 Upregulated HK2 in the Process of the ASP-AMP Promoting Liver Regeneration

**Figure 7:**
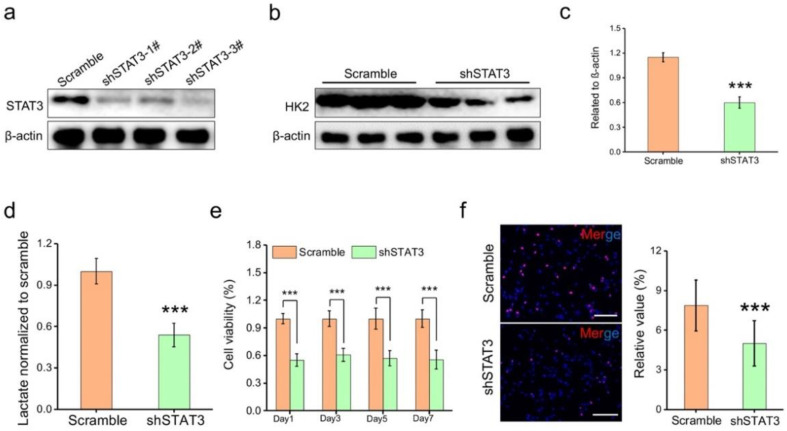
ASP-AMP accelerated HK2-assisted glycolysis via JAK2/STAT3 activation to enhance liver regeneration. (**a**) Efficiency of knockdown of STAT3 in NCTC clone 1469 cells. (**b**) HK2 was analyzed by Western blot in STAT3 knockdown hepatocytes (*n* = 3). (**c**) Densitometric analysis of protein expression related to β-actin. (**d**) Lactate in supernatant of STAT3 knockdown hepatocytes (*n* = 3). (**e**) The cell viability of STAT3 knockdown NCTC clone 1469 cells treated with ASP-AMP for 7 days (*n* = 3). (**f**) Click-iT EdU proliferation staining and relative positive EdU staining analysis of STAT3 knockdown NCTC clone 1469 cells treated with ASP-AMP for 3 days (Scale bar = 100 µm). *** *p* < 0.001.

To evaluate the relationship between STAT3- and HK2-involved glycolysis in liver regeneration promoted by ASP-AMP, STAT3 was knocked down in NCTC clone 1469 cells (shSTAT3#3) (Figure 7a). The knockdown of STAT3 decreased the protein expression of HK2 (Figure 7b,c) and diminished the lactate secretion in the supernatant after ASP-AMP intervention (*p* < 0.001, Figure 7d). Subsequently, the effects on cell proliferation were analyzed. Cell viability and the EdU assay displayed that cell proliferation was significantly inhibited in STAT3 knockdown NCTC clone 1469 cells (*p* < 0.001, Figure 7e,f).

## 3. Discussion

Improving liver regeneration after hepatectomy has clinically proven difficult. Although surgical strategies including portal vein embolization/ligation and associated liver partition and portal vein ligation for staged hepatectomy have been proven to be effective in liver regeneration, applications are limited due to strict inclusion criteria, severe complications, and high medical costs [23]. Agents including amiodarone [24], thalidomide [25], etanercept [26], and bicyclol [27] have been reported to enhance liver regeneration in animal models; however, they were rarely applied in clinical practice due to their side effects (kidney damage, gut reaction, perception abnormalities, or perception barriers, etc.) and toxicity. Physiologically, the cell proliferative response to hepatectomy lasted for 72 h, reaching a peak at 24–36 h, and then increasingly shifted to a termination phase [28]. Rapid administrations to regulate the proliferative process during the initial 72 h were of significance in enhancing liver regeneration [29].

Simultaneously, ASP and AMP—the main active polysaccharides in DBD—obtained essential implications in promoting regeneration [30]. By means of different extraction or structural synthesis processes, bioactive polysaccharides from plants have been tested for the biological engineering and regeneration of practically all tissues [6,31,32]. For instance, hyaluronic acid was proven to participate in articular pathologies, skin remodeling, vascular prosthesis, adipose tissue engineering, and nerve reconstruction [33]. Decorin accelerated liver regeneration after partial hepatectomy in fibrotic mice [34]. As for ASP, it has been proven to exert a hepatoprotective effect in CCl_4_-induced hepatic dysfunction and tissue damage in mice [35]. Hepatic toxicity caused by 5-fluorouracil could be antagonized by ASP via apoptosis reduction through the Nrf2 pathway [36]. AMP reduced endoplasmic reticulum stress and restored glucose homeostasis in the liver [37]. Furthermore, the hepatoprotective effect of ASP-AMP was observed in CCl_4_-induced intoxication in mice, and the effect was stronger than that of the respective use of ASP or AMP [14]. Based on the evidence, this study verified that ASP-AMP exerted a significant promoting effect on liver regeneration in vitro and in vivo, as well as hepatoprotection after liver resection, especially during the first 72 h. This provides evidence that supplementation of Qi and blood via ASP and AMP facilitated the regenerative capacity of the liver. Meanwhile, the promoting effect of ASP and AMP on cell proliferation was observed, which was within an effective range of concentrations from 12.5 to 3000 µg/mL. Importantly, these results support the safety of ASP and AMP application for further clinical use.

Major hepatectomy in mice metabolically led to a rapid fall in blood glucose levels in terms of a dramatic reduction in glycogen stores and gluconeogenic capacity [38]. However, the restoration of liver mass depended on increased energy production, which was indispensable for the recovery of liver function [39]. Increased hepatic glycometabolism was observed in an improved liver regeneration after fatty liver resection [40]. Thus, the metabolic process might be regulated for enhanced liver regeneration within the 72 h proliferative phase. With the promoting effect of ASP-AMP, metabonomics suggest that differential metabolites are enriched in the glycolysis process with related metabolites including beta-d-fructose, 6-phosphate, and lactate. As glucose was metabolized into lactate via glycolysis in the cytoplasm, the increased lactate levels in supernatant and serum verified the enhanced glucose mobilization in the initial period of liver regeneration. This was also consistent with a previous report which indicated that glycolysis actively participated in regulating the termination of liver regeneration [41]. Furthermore, we observed overexpression of HK2 in the transcription and protein level caused by ASP-AMP treatment, and conversely the knockdown of HK2 elicited decreased lactate secretion and suppressed cell proliferation in vitro and in vivo. As an essential rate-limiting enzyme in glycolysis, HK2 has been reported to be actively involved in glycolysis-related liver diseases, especially in hepatocellular carcinoma [42,43]. As for liver regeneration, the prolonged regaining of liver weight after hepatectomy could be observed to be parallel with decreased activities of the HK2-related glycolytic pathways by microarray analysis [44]. Therefore, this indicates that ASP-AMP mediates glycolysis via the regulation of HK2.

Regarding ASP-AMP’s promoting role in liver regeneration, the specific mechanism remains to be explored. Through predictions of network pharmacology and further verification, we focused on the JAK2/STAT3 pathway activated by ASP-AMP. The JAK2/STAT3 pathway and the following cascade was considered to be a critical pathway in priming the hepatocytes for proliferation [45]. JAK2 is one of the JAKs which have been identified as being widely expressed in human cells and have molecular masses ranging from 120 to 140 kDa [46]. JAK2 mediates the activation of STAT3 in cells exposed to formulations. In detail, the proliferation process involved IL-6R activation and the engagement of JAK protein-bound gp130, the promotion of which resulted in phosphorylating STAT3, causing an expression of multiple target genes important for hepatocyte proliferation such as MYC, MCL1, and PCNA [3,47]. The JAK2/STAT3 activation played a pivotal role in the acceleration of liver hypertrophy in a rat hepatectomy model [48]. The suppressed mitogenic JAK2/STAT3 pathway inhibited cell cycle progression and constrained the ability of partial hepatectomy to stimulate hepatocyte replication [49]. These observations were consistent with our observation that the activation of the STAT3 pathway contributed to cell proliferation in a liver graft [50]. Simultaneously, the JAK2/STAT3 pathway has also been proven to be positively associated with glycolysis which was regulated in the malignant progression of tumors [51,52]. These above results support the assertion that JAK2/STAT3 plays a role in liver regeneration via glycolysis. Although evidence has indicated that ASP or AMP indirectly regulate the JAK2/STAT3 pathway in various diseases [53,54], the combined effect of ASP-AMP on liver regeneration is so far unreported, especially in metabolic pathways.

Based on the role of JAK2/STAT3 in glycolysis and liver regeneration, the regulatory effect between STAT3 and HK2 should be further clarified. Previous reports indicated that the upregulation of STAT3/HK2 contributed to the activation of dendritic cells by leptin-induced glycolytic metabolism [55], and circular RNA circCUL3 eliciting the Warburg effect of gastric cancer [56]. Meanwhile, suppressed proliferation in hepatocellular carcinoma cells or the inhibition of glycolysis was achieved by the blocking of the JAK2/STAT3 pathway [57,58]. Furthermore, STAT3 was reported to bind to the promoter of the HK2 gene to regulate proliferation in gastric epithelial cells [59]. In the therapy of porcine delta coronavirus by selenomethionine, the STAT3 could repress miR-125b-5p-1 expression, which targets and inhibits the expression of HK2 [60]. In line with these reports, we observed that by the knockdown of STAT3 in hepatocytes, HK2 expression was downregulated accordingly, followed by inhibited lactate secretion and cell proliferation. These results imply that the activation of STAT3 could regulate glycolysis and hepatocytes proliferation via HK2.

## 4. Materials and Methods

### 4.1. Cell Culture and Reagent

Normal liver cell line NCTC clone 1469 and BRL-3A cells were purchased from the Shanghai Institute of Cell Biology at the Chinese Academy of Sciences (Shanghai, China) and cultured in DMEM supplemented with 10% fetal bovine serum (BSA, 1099-141, Thermo Fisher Scientific, Inc., Waltham, MA, USA), 1 × 10^5^ U/L penicillin and 100 mg/L streptomycin (15140122, Thermo Fisher Scientific, Inc., Waltham, MA, USA) in a humidified atmosphere with a 5% CO_2_ incubator at 37 °C. Trypsin-EDTA (0.05%) (Thermo Fisher Scientific, Inc., Waltham, MA, USA) was used for cell passages. Cells were authenticated by STR profiling, and experiments were performed from 5 passages. shRNAs targeting HK2 or STAT3 were designed by OBiO Biotechnology Co. LTD (Shanghai, China). Three shRNAs targeting the coding sequence of mRNA were inserted into the pLKO.1 vector. The efficacy of each shRNA was assessed by Western blot of the NCTC clone 1469 cells, which were infected with the virus for 3 days. The shRNA with strongest knockdown efficacy was used for further experiments. ASP (DSTDDO28301) and AMP (DSTDH010901) were purchased from Desite Biotechnology Co., LTD (Chengdu, China). The information regarding ASP and AMP, including identification, ingredients, and physicochemical properties, etc., can be seen in http://www.028desite.com (accessed on 6 December 2021). Ruxolitinib (SD4740) and 2-deoxy-d-glucose (2DG, ST1024) were obtained from Beyotime Biotechnology (Shanghai, China).

### 4.2. Cell Viability

NCTC clone 1469 and BRL-3A cell lines were inoculated into 96-well plates with a concentration of 1 × 10^4^ cells per well and incubated with a variety of concentrations (12.5 μg/mL, 25 μg/mL, 50 μg/mL, 100 μg/mL, 200 μg/mL, 400 μg/mL, 800 μg/mL, 1600 μg/mL, and 3200 μg/mL) of ASP and AMP for 7 days. Cell viability was determined by a AlamarBlue^®^ Cell Viability Assay Kit (AB, DAL1100, Invitrogen Corporation, Waltham, MA, USA) on day 1, day 3, day 5, and day 7. The cells were supplemented with AB solution (medium199: fetal bovine serum: AB = 8:1:1 *v*/*v*) after the removal of the medium and PBS washing. For 4 h of incubation, the absorbance (optical density, OD) at 570 nm was measured and calculated according to the formula: Cell viability (%) = (OD experimental − OD blank)/(OD control − OD blank) × 100%. Experiments were performed in triplicate.

### 4.3. Click-iT Plus EdU Proliferation Staining

ASP (20 μg/mL) and/or AMP (100 μg/mL) were added to NCTC clone 1469 cells, and ASP (160 μg/mL) and/or AMP (800 μg/mL) were added to BRL-3A cells for 3 days’ culture in 12-well plates. The EdU working solution (Beyotime Biotechnology, Shanghai, China) was added to the culture medium to obtain a final concentration of 10 μM. The cells were incubated with the solution for 2 h in an incubator at 37 °C. After removal of the solution, cells were treated with paraformaldehyde (1.5 M) for 15 min. Cells were then washed in PBS for 15 min, followed by 0.5% Triton-X 100 in PBS for 10 min. Click Additive Solution was prepared according to the manufacturer’s instructions and added to each well, which was incubated for 30 min in the dark. Then, 5 μg/mL Hoechst 333,425 was added to the wells, and they were incubated for additional 30 min. During every step, cells were washed twice with 3% BSA in PBS. Finally, cells were examined using the Zeiss fluorescence microscope. Four views of each well were randomly recorded with triplicate samples (*n* = 3). The quantification of blue and red points was performed by ImageJ (v 1.53, National Institutes of Health, Stapleton, NY, USA).

### 4.4. Experimental Animals

The study was approved by the Animal Research Ethics Committee of Chengdu University of Traditional Chinese Medicine (Chengdu, China) and complied with the Guidelines for Animal Experiments. Thirty-six Balb/c mice aged 5 weeks old and weighing 18–22 g were purchased from Chengdu Dashuo Biotechnology Co., Ltd. (Chengdu, China; license no. SCXK 2008-24). All mice were maintained on a 12/12 h light/dark cycle, allowed free access to water and food, and acclimated for at least 5 days prior to surgery. Mice in the Sham group underwent the procedures of opening and closing the abdomen. Mice in the control group underwent 70% liver resection without any other treatment. Mice in the ASP-AMP group were orally administrated ASP (250 mg/kg/day) and AMP (1 g/kg/day) for 7 days after 70% hepatectomy. Mice in the ASP-AMP+2DG group were injected intraperitoneally with 2-DG (a hexokinase inhibitor, 1 g/kg, Sigma, Arklow, Ireland) on days 1, 2, 3, and 6 using sterile saline as a vehicle, based on the treatment in the ASP-AMP group. Inhalation of CO_2_ (20% of the displacement volume per minute) was used for euthanasia. Harvested liver tissues were fixed in 4% paraformaldehyde for 24 h at room temperature and stored in 75% ethanol at 4 °C for further histology and immunohistochemistry (IHC) analysis.

### 4.5. Seventy Percent Partial Hepatectomy in a Murine Model

For the mouse model of partial hepatectomy, 2/3 of the liver was resected according to the previous description [61]. Briefly, mice were anesthetized with 2% isoflurane and 2 L/min oxygen flow to maintain anesthesia by isoflurane. A midline abdominal skin and muscle incision was created to expose the xiphoid process. The 4-0 silk thread was placed on the base of the left lateral lobe and the lobe was ligated over the top of it. The tied lobe was curved above the suture using microsurgery. Similarly, the median hepatic lobes were then ligated and resected. Finally, after closing the abdomen, the skin surrounding the suture was wiped with betadine, and mice were placed on a warming pad for recovery.

### 4.6. Enzyme-Linked Immunosorbent Assay (ELISA)

The whole blood of sacrificed mice was kept at room temperature for 2 h and centrifuged at 4000 rpm for 10 min to collect serum. Serum ALT (JL20911, Jonln Co., Ltd., Shanghai, China), AST (C010-2-1, Nanjing Jiancheng Bio Co., Ltd., Nanjing, China), and total bilirubin (C019-1-1, Nanjing Jiancheng Bio Co., Ltd., Nanjing, China). According to the instructions, the concentration gradient dilution standard was added to the 96-well plate (100 μL per well), 100 μL of mouse serum was then added to the 96-well plate in turn, and each sample was placed into 3 duplicate wells, which were incubated at 37 °C for 1 h. After discarding the liquid, 100 μL per well of biotinylated antibody was directly added without washing and incubated at 37 °C for 1 h. Then, the samples were washed 3 times with 1 × wash solution. After this, 100 μL per well of enzyme conjugate working solution was added and incubated at 37 °C for 30 min. Subsequently, 90 μL per well of substrate was added, and incubated at 37 °C for 15 min, followed by 10 μL per well of stop solution. We immediately measured the absorbance of the solution at 450 nm on a microplate (Multiskan GO, Thermo Fisher Scientific, Inc., Waltham, MA, USA).

### 4.7. Untargeted Metabolomics by Liquid Chromatography Coupled Mass Spectrometry (MS/MS)

See details in Methods S1.

### 4.8. Lactate Measurement

For lactate measurement in serum, the whole blood of treated mice was firstly collected and centrifuged at 4000 rpm for 10 min to obtain the serum. For the analysis of lactate in cell cultures, cells were seeded at approximately 3 × 10^5^ in 6-well plates and treated with different concentrations of ASP and/or AMP for 3 days. After centrifugation, the culture medium and cells were collected. The lactate content was estimated using the l-lactate assay kit (Jiancheng Bioengineering, Nanjing, China) according to the manufacturer’s protocol. Lactate dehydrogenase catalyzed the conversion of lactate to pyruvic acid, and the absorbance at 530 nm was determined spectrophotometrically after the addition of a color developing agent, which showed a linear relationship with lactate content. Three replicates were performed, and the experiments were repeated three times for accuracy.

### 4.9. Western Blot

Total cell protein was collected (KGP2100, KeyGEN BioTECH, Beijing, China), and its quantification was performed using the BCA (cat. no. KGP902, KeyGEN BioTECH, Beijing, China) method. Proteins were separated by SDS-PAGE and transferred through PVDF membranes. After blocking the membrane with 5% BSA for 2 h at room temperature, membranes were incubated overnight at 4 °C with one of the following primary antibodies: rabbit monoclonal anti-STAT3 (ab68153, Abcam, Cambridge, UK), rabbit monoclonal anti-phospho STAT3 (ab32143, Abcam, Cambridge, UK), rabbit monoclonal anti-Janus kinase 2 (JAK2, ab108596, Abcam, Cambridge, UK), rabbit monoclonal anti-phospho JAK2 (ab32101, Abcam, Cambridge, UK), rabbit monoclonal anti-proliferating cell nuclear antigen (PCNA, ab92552, Abcam, Cambridge, UK), rabbit monoclonal anti-MYC (ab32072, Abcam), mouse monoclonal anti-P53 (ab90363, Abcam, Cambridge, UK), rabbit monoclonal anti-β-actin (Bsm-33036M, Biosynthesis, Beijing, China), rabbit monoclonal anti-HK2 (ab209847, Abcam, Cambridge, UK), and rabbit monoclonal anti-glucose transporter 1 (GLUT1, ab115730, Abcam, Cambridge, UK). All antibodies were diluted at 1:1000 in TBST (T1086, Solarbio, Beijing, China). After washing with PBS, membranes were incubated with secondary antibodies, including goat anti-mouse IgG antibody (bs-0296G, Bioss, Beijing, China) and goat anti-rabbit IgG antibody (bs-0295G; Bioss, Beijing, China), which were diluted to 1:4000 by TBST, for 1 h at room temperature. After washing off the excess secondary antibody, protein intensity was determined with Clarity Western ECL Substrate (Bio-Rad Laboratories Co. Ltd., Hercules, CA, USA) and measured by Image Lab software (5.2.1 Version, Bio-Rad Laboratories Co. Ltd., Hercules, CA, USA). Proteins were quantified by densitometric analysis of ImageJ (version 1.8.0.172, Bethesda, MA, USA).

### 4.10. Histology and Immunohistochemistry (IHC) Analysis

Murine liver tissues were collected, fixed in 4% paraformaldehyde for 48 h, dehydrated, and embedded in paraffin. The paraffin tissue blocks were cut into 4 μm-thick sections, deparaffinized with xylene and passed through graded alcohol (10009218, ChengFeng Co., Ltd., Hangzhou, China). To detect the expression of HK2 and Ki-67 in liver tissue, sections were incubated with anti-HK2 antibody (1:200, ab209847, Abcam, Cambridge, UK) or anti-Ki67 antibody (1:200, bs-2130R, Bioss, Beijing, China). Sections were incubated overnight at 4 °C and rinsed 3 times with 1 × PBS for 5 min each time. Reagents were added according to the instructions of the secondary antibody kit (sp-9000; ZSGB-Bio, Beijing, China), and finally DAB (ZLI-9018; ZSGB-Bio, Beijing, China) were added for a 2 min reaction. Four views were randomly collected from each section, and images were acquired using an optical microscope (DM3000, Leica Co., Ltd., Weztlar, Germany).

### 4.11. Real-Time Quantitative PCR Analysis

Total cellular RNA was extracted by using Trizol Reagent (Invitrogen, Thermo Fisher Scientific, Inc., Waltham, MA, USA). Reverse transcription was performed using PrimeScript (Takara Bio, Inc., Nojihigashi Kusatsu, Japan), first removing gDNA by using the following conditions: 42 °C for 2 min. The conditions used for reverse transcription were as follows: 37 °C for 15 min and 85 °C for 5 s. The cDNA was subjected to PCR by adding SYBR Green (Takara Bio, Inc., Nojihigashi Kusatsu, Japan), and the sequences of the primers used are shown in Table 1. Real-time quantitative PCR detection was performed by using a CFX96 Deep well (Bio-Rad Laboratories, Inc., Hercules, CA, USA) under the following cycling conditions: 95 °C for 30 s, 95 °C for 5 s, and 60 °C for 30 s, with 40 cycles. Experiments normalized the mRNA expression of each gene with β-actin as an internal control. All samples were repeated three times. Finally, the relative gene expression results were calculated using the 2^−ΔΔCt^ method.

### 4.12. Network Pharmacology and Bioinformatics

See details in Methods S2.

### 4.13. Statistical Analysis

Statistical analysis was performed using SPSS version 22.0 (IBM, Corp., Armonk, NY, USA). All the data are expressed as mean ± standard deviation. One-way ANOVA or unpaired two-tail Student’s t-test was used to determine differences among groups. A statistically significant difference was considered significant at *p* = 0.05, * *p* < 0.05, ** *p* < 0.01, and *** *p* < 0.001.

## 5. Conclusions

The present investigations demonstrated that ASP-AMP acted as a promoting factor for liver regeneration. ASP-AMP intervention in hepatocytes enhanced proliferation by accelerating HK2-associated glycolysis. Moreover, ASP-AMP-induced glycolysis involved liver regeneration by JAK2/STAT3/HK2 signaling. These findings demonstrate that ASP and AMP, as natural agents, are feasible and experimentally recommended in the treatment of liver regeneration in patients who undergo hepatectomy. To impel the use of ASP-AMP in clinic therapy, the pharmacokinetics and toxicity should be further investigated. In addition, several missing links remain to be elucidated in future studies, including the molecular mechanism responsible for the ASP-AMP-mediated upregulation of JAK2 and the STAT3-induced HK2 activation.

## Figures and Tables

**Table 1:** Primers which have been used for real time PCR.

Primer Name	Sequence 5′-3′	Product Length
HK2	Forward: ATGATCGCCTGCTTATTCACG	110 bp
	Reverse: CGCCTAGAAATCTCCAGAAGGG	
LDHA	Forward: ACATTGTCAAGTACAGTCCACAC	114 bp
	Reverse: TTCCAATTACTCGGTTTTTGGGA	
GAPDH	Forward: TGACCTCAACTACATGGTCTACA	85 bp
	Reverse: CTTCCCATTCTCGGCCTTG	
PFKFB3	Forward: CAACTCCCCAACCGTGATTGT	83 bp
	Reverse: TGAGGTAGCGAGTCAGCTTCT	
PKM2	Forward: CGCCTGGACATTGACTCTG	135 bp
	Reverse: GAAATTCAGCCGAGCCACATT	
GLUT1	Forward: TCAAACATGGAACCACCGCTA	123 bp
	Reverse: AAGAGGCCGACAGAGAAGGAA	
C-JUN	Forward: ACTCGGACCTTCTCACGTC	110 bp
	Reverse: GGTCGGTGTAGTGGTGATGT	
EGFR	Forward: ATGAAAACACCTATGCCTTAGCC	83 bp
	Reverse: TAAGTTCCGCATGGGCAGTTC	
VEGFA	Forward: GCACATAGAGAGAATGAGCTTCC	105 bp
	Reverse: CTCCGCTCTGAACAAGGCT	
IL-6	Forward: CTGCAAGAGACTTCCATCCAG	131 bp
	Reverse: AGTGGTATAGACAGGTCTGTTGG	
STAT3	Forward: CACCTTGGATTGAGAGTCAAGAC	112 bp
	Reverse: AGGAATCGGCTATATTGCTGGT	
JAK2	Forward: GGAATGGCCTGCCTTACAATG	108 bp
	Reverse: TGGCTCTATCTGCTTCACAGAAT	
MAPK1	Forward: GGTTGTTCCCAAATGCTGACT	84 bp
	Reverse: CAACTTCAATCCTCTTGTGAGGG	
SRC	Forward: TTTGGCAAGATCACTAGACGGG	111 bp
	Reverse: GAGGCAGTAGGCACCTTTTGT	
INS	Forward: CACTTCCTACCCCTGCTGG	177 bp
	Reverse: ACCACAAAGATGCTGTTTGACA	
TNF	Forward: CAGGCGGTGCCTATGTCTC	89 bp
	Reverse: CGATCACCCCGAAGTTCAGTAG	
β-actin	Forward: GGCTGTATTCCCCTCCATCG	154 bp
	Reverse: CCAGTTGGTAACAATGCCATGT	

## Data Availability

The data presented in this study are available upon request from the corresponding author.

## Supplementary Materials

The following supporting information can be downloaded at: https://www.mdpi.com/article/10.3390/molecules27227890/s1, Method S1: Untargeted metabolomics by liquid chromatography coupled mass spectrometry (MS/MS); Method S2: Network pharmacology and bioinformatics; Figure S1: *Angelica sinensis* polysaccharide (ASP) and *Astragalus membranaceus* polysaccharide (AMP) promoted the proliferation of BRL-3A; Figure S2: Combined use of both ASP and AMP enhanced the proliferation of BRL-3A; Figure S3: Volcanic map of differential metabolites; Figure S4: Potential targets analysis of drugs (ASP and AMP) and liver regeneration.

## Sample Availability

Samples of ASP and AMP are available from the corresponding author.
