# Supplementary material for: Angelica sinensis Polysaccharide and Astragalus membranaceus Polysaccharide Accelerate Liver Regeneration by Enhanced Glycolysis via Activation of JAK2/STAT3/HK2 Pathway
Source: Molecules. 2022 Nov 15;27(22):7890. doi: 10.3390/molecules27227890 (PMC9695464; doi:10.3390/molecules27227890)
Supplement: Supplementary file 1 [file molecules-27-07890-s001.zip › molecules-2015111-supplementary.pdf]

1 Supplementary methods

1.1 Untargeted metabolomics by liquid chromatography coupled mass spectrometry (MS/MS)

Cultured cells were treated with AsP and AnP for 3 days and centrifuged at 1200 rpm for 5 min. 1 × 10^7 cells each of 12 samples (n=6) of NCTC clone 1469 were collected. For tissue samples, the 7 days of AsP and AnP administrated mice were sacrificed, and 1 g each of 16 samples (n=8) of liver tissues was obtained. The samples were frozen in liquid nitrogen for storage, and transported with dry ice to Beijing Tiangen Technology Co., Ltd. for analysis.

1.1.1 Metabolites Extraction

The samples were individually grounded with liquid nitrogen and the homogenate was resuspended with prechilled 80% methanol and 0.1% formic acid by well vortexing. The samples were incubated on ice for 5 min and then centrifuged at 15000 rpm, 4℃ for 5 min. And some of supernatant was diluted to final concentration containing 53% methanol by LC-MS grade water. The samples were subsequently transferred to a fresh Eppendorf tube and then centrifuged at 15000g, 4℃ for 10 min. Finally, the supernatant was injected into the LC-MS/MS system for analysis. Liquid sample (100 μL) and prechilled methanol (400 μL) were mixed by well vortexing.

1.1.2 UHPLC-MS/MS Analysis

LC-MS/MS analyses were performed using a Vanquish UHPLC system (Thermo Fisher) coupled with an Orbitrap Q Exactive series mass spectrometer (Thermo Fisher). Samples were injected into an Hyperil Gold coulum (100 mm × 2.1 mm, 1.9 μm) using a 16-min linear gradient at a flow rate of 0.2mL/min. The eluents for the positive polarity mode were eluent A (0.1% FA in Water) and eluent B (Methanol). The eluents for the negative polarity mode were eluent A (5 mM ammonium acetate, pH 9.0) and eluent B (Methanol). The solvent gradient was set as follows: 2% B, 1.5 min; 2-100% B, 12.0 min; 100% B, 14.0 min; 100-2%B, 14.1 min; 2% B, 17 min. Q Exactive series mass spectrometer was operated in positive/negative polarity mode with spray voltage of 3.2 KV, capillary temperature of 320℃, sheath gas flow rate of 35 arb and aux gas flow rate of 10 arb.

1.1.3 Database search

Raw data files generated by UHPLC-MS/MS were processed by Compound Discoverer 3.1 (CD3.1, Thermo Fisher) to perform peak alignment, peak picking, and quantitation for each metabolite. The main parameters were set as follows: retention mass tolerance, 5ppm; signal intensity tolerance, 30%; signal/noise ratio, 3; and minimum intensity, 100000. After that, peak intensities were normalized to the total spectral intensity. The normalized data was

used to predict the molecular formula based on additive ions, molecular ion peaks and fragment ions. And the peaks were matched with the mzCloud (https://www.mzcloud.org/), mzVault and MassList database to obtain the accurate qualitative and relative quantitative results. Statistical analysis were performed using the statistical software R (R version R-3.4.3), Python (Python 2.7.6 version) and CentOS (CentOS release 6.6).

1.1.4 Data Analysis

The metabolites were annotated with the KEGG database (http://www.genome.jp/kegg/), HMDB database (http://www.hmdb.ca/) and Lipidmaps database (http://www.lipidmaps.org/). Principal components analysis and Partial least squares discriminant analysis were performed by metaX (a flexible and comprehensive software for processing metabolomics data). We applied univariate analysis (t-test) to calculate the statistical significance (*p*-value). The metabolites with VIP > 1 and P-value< 0.05 and fold change≥ 2 or FC≤ 0.5 were considered to be differential metabolites. Volcano plots were used to filter metabolites of interest which based on Log_2 (FC) and $-log_{10}$ (P-value) of metabolites. For clustering heat maps, the data were normalized using z-scores of the intensity areas of differential metabolites and were plotted by Pheatmap package in R language. The correlation between differential metabolites were analyzed by cor. in R language (method = pearson). Significant correlation differential metabolites were calculated by cor.mtest in R language. *p*-value < 0.05 was considered as statistically significant and correlation plots were plotted by corrplot package in R language. The functions of the metabolites and metabolic pathways were studied using the KEGG database. The metabolic pathway enrichment of differential metabolites were performed when ratio reached x/n > y/N. When *p*-value of metabolic pathway < 0.05, metabolic pathway were considered as statistically significantly enriched.

1.2 Network pharmacology and bioinformatics

1.2.1 Data preparation

Traditional Chinese Medicine System Pharmacology Database (TCMSP, https://www.tcmspw.com/index.php), Pub Chem Database (http://pubchem.ncbi.nlm.nih.gov) and swiss target prediction Database (http://www.swisstargetprediction.ch/) were used to collect the potential targets of ASP and AMP. Liver regeneration related targets from GeneCards database (https://www.genecards.org/), OMIM database (https://omim.org/), NCBI database (http://www.ncbi.nlm.nih.gov) and Pharm GKB database (www.pharmgkb.org) were collected using "Liver regeneration" as keyword, and then converted the target names to the gene names through the Uniprot database (https://www.uniprot.org/). We screened out

disease targets that appeared at least twice, and used TBtools1.038 (https://github.com/CJ-Chen/TBtools/releases) to intersect with the therapeutic targets of ASP and AMP to obtain potential therapeutic targets.

1.2.2 Protein-protein interaction (PPI) networks construction

These potential therapeutic targets were then put in STRING tools (https://string-db.org/). After that, import the result into CytoScape3.8.2 (http://cytoscape.org/) for PPI network visualization.

1.2.3 Enrichment analysis

Potential therapeutic targets were imported into the Database for Annotation, Visualization and Integrated Discovery (DAVID) 6.8 (https://david.ncifcrf.gov/) to obtain Gene ontology (GO) and KEGG enrichment analysis to further reveal the potential mechanism of astragalus polysaccharide and angelica polysaccharide in Liver regeneration. The plots of GO enrichment were then performed in bioinformatics (https://www.bioinformatics.com.cn), and KEGG pathway enrichment were imported into CytoScape3.8.2 for ClueGo plug-in.

2 Supplementary figures

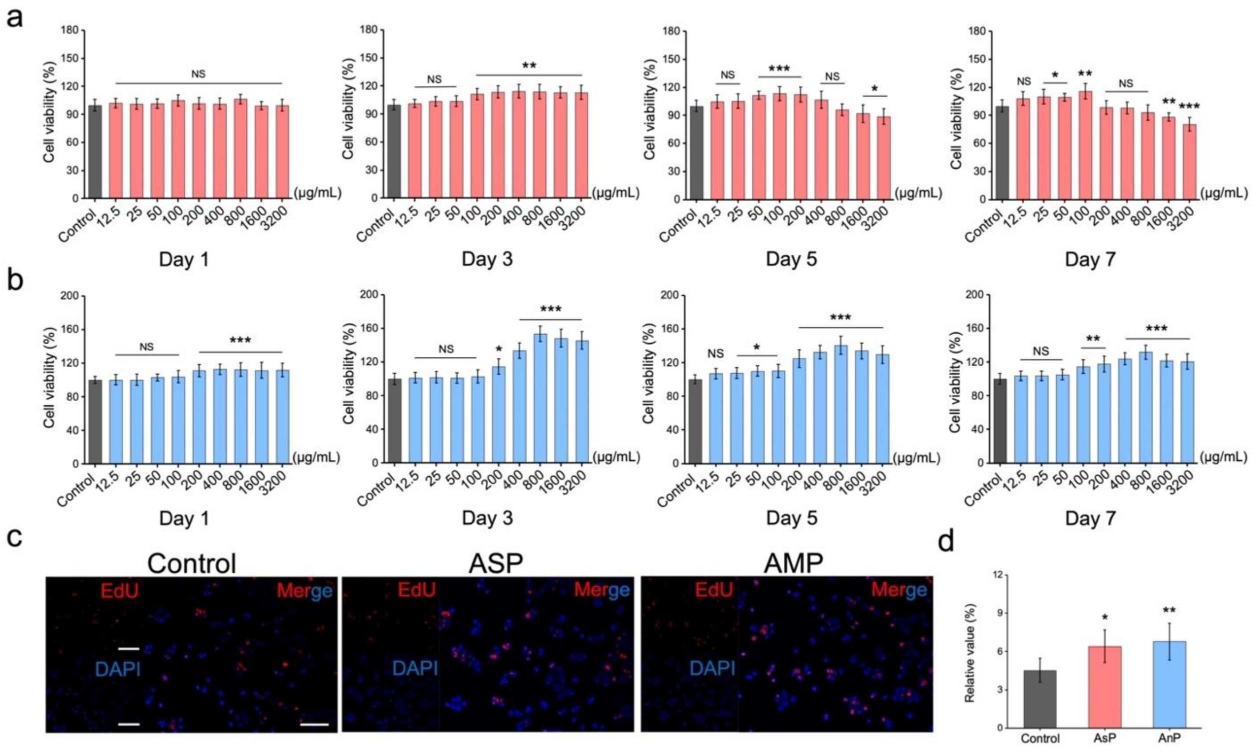

Figure S1. *Angelica sinensis* polysaccharide (ASP) and *Astragalus membranaceus* polysaccharide (AMP) respectively promoted proliferation of BRL-3A. The cell viability of hepatocytes with various concentrations of ASP (**a**) and AMP (**b**) for 7 days' incubation, which was determined by Alamar Blue Cell Viability Reagent. (**c**) Click-iT EdU proliferation staining of hepatocytes respectively treated with ASP or AMP for 3 days (Scale bar = 100 μm). (**d**) Analysis of relative expression rates of positive EdU staining. $^{*}p < 0.05$, $^{**}p < 0.01$, and $^{***}p < 0.001$.

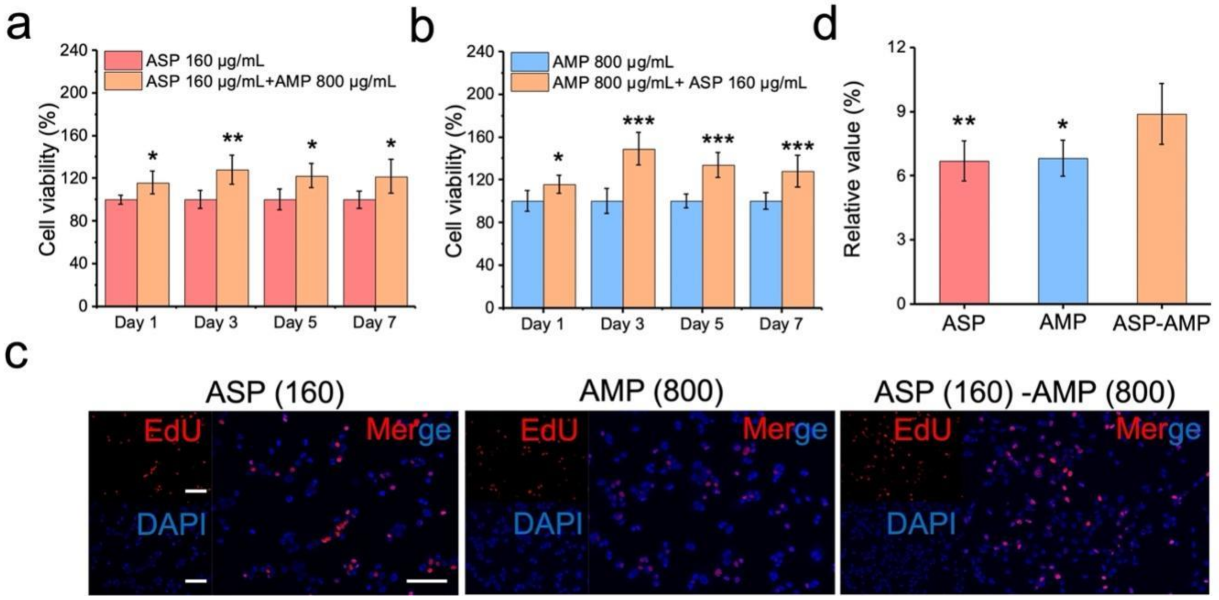

Figure S2. Combined use of both ASP and AMP enhanced the proliferation of BRL-3A. The cell viability of hepatocytes with ASP-AMP normalized to separate use of ASP (**a**) or AMP (**b**) for 7 days. (**c**) Click-iT EdU proliferation staining of hepatocytes treated with ASP and AMP (Scale bar = 100 μm). (**d**) Relative expression rates of positive EdU staining. * $p < 0.05$, ** $p < 0.01$, and *** $p < 0.001$.

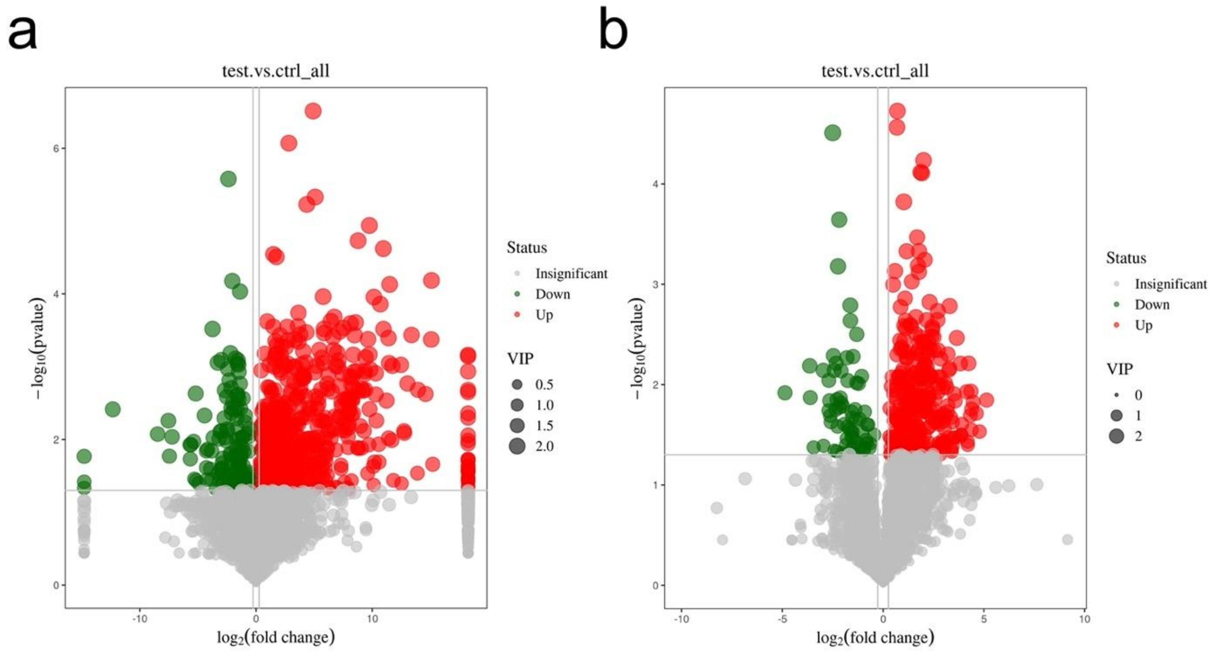

Figure S3. Volcanic map of differential metabolites of NCTC clone 1469 cells (n = 6) (**a**) and liver tissue (n = 8) (**b**) after hepatectomy when treated with ASP-AMP. Grey was insignificant, green was down-regulated, and red was up-regulated.

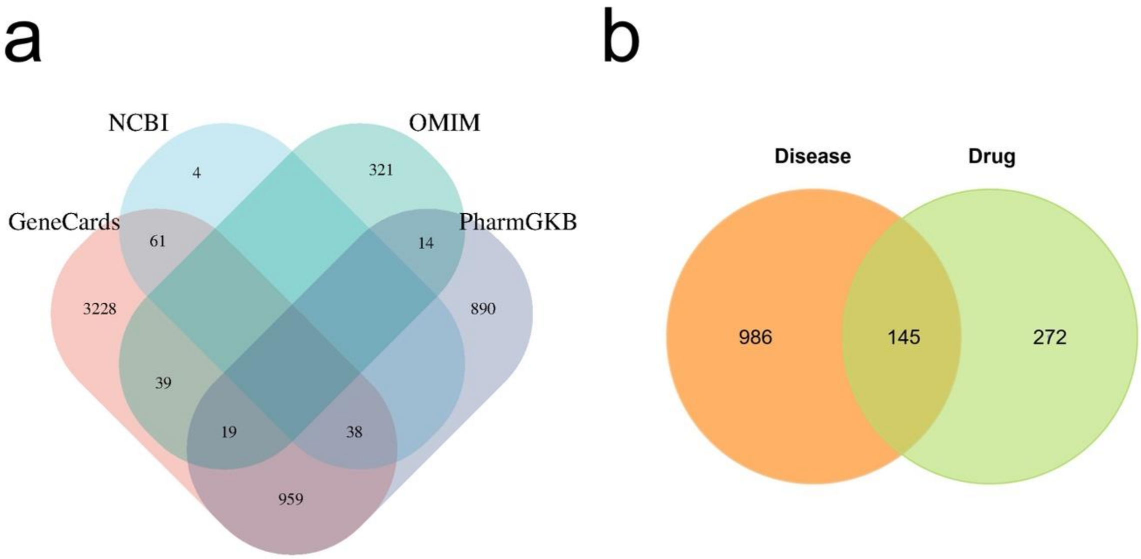

Figure S4. Potential targets analysis of drugs (ASP and AMP) and liver regeneration. (**a**) Venn diagram of targets in liver regeneration. (**b**) Venn diagram of intersecting targets of drugs (ASP and AMP) and liver regeneration.
